# Memantine for the patients with mild cognitive impairment in Parkinson’s disease: a pharmacological fMRI study

**DOI:** 10.1186/s12883-022-02699-x

**Published:** 2022-05-13

**Authors:** Shoji Kawashima, RCIP-Nagoya Study Group, Yoko Shimizu, Mitsuya Horiba, Yoshino Ueki, Ryohei Akanabe, Hirohito Kan, Haruaki Kasai, Hiroshi Kunitomo, Satoshi Tanaka, Takenari Toyota, Shoji Kawashima, Masayuki Mizuno, Kenji Okita, Noriyuki Matsukawa, Noriyuki Matsukawa

**Affiliations:** Department of Neurology and Neuroscience, Nagoya City University Graduate School of Medical Science, 1 Kawasumi, Mizuho-ku, Nagoya, 467-8601 Japan

**Keywords:** Mild cognitive impairment, Parkinson’s disease, Dementia, Functional MRI, Working memory, N-back test, Memantine, Daytime sleepiness, Superior frontal gyrus

## Abstract

**Background:**

Mild cognitive impairment in Parkinson’s disease (PD-MCI) is associated with an increased risk of cognitive decline. PD-MCI is characterized by impairments in executive function and visuospatial recognition. The visuospatial n-back test is useful for assessing both domains. The 0-back test reflects visuospatial recognition, while the 1-back and 2-back tests reflect working memory. Cholinesterase inhibitors are effective in the treatment of PD-MCI and dementia in PD (PDD). Although some studies have reported the efficacy of memantine for PDD, the therapeutic efficacy of memantine in patients with PD-MCI remains uncertain.

**Methods:**

This study aimed to investigate the effects of memantine on brain function in patients with PD-MCI, using a randomized double-blinded crossover protocol and functional MRI (fMRI). Ten patients who completed 16 weeks of follow-up were included. They were randomly assigned to either the memantine or placebo. Patients in the memantine group received 5 mg/day of memantine in the first week. The memantine dose was increased by 5 mg/day per week, until a final dose of 20 mg/day. Patients in the placebo group received the placebo following the same regimen as memantine. After the intervention, they underwent a 4 weeks washout period. Following the crossover protocol, a second intervention was conducted after the washout period. In each intervention, fMRI and neuropsychological tests were performed at the maximum dose period. Comparing the memantine and placebo groups, we investigated difference in the brain regions using the visuospatial n-back test.

**Results:**

There were no significant regions enhanced by memantine comparing with placebo at any load of n-back tests. In contrast, exploring regions reduced by memantine, we found significant reduction of activations within right lingual gyrus and left superior frontal gyrus in comparison between 2-back and 0-back test. A number of correct answers of the 2-back test and time to complete Trail Making Test-A were worse during memantine intervention.

**Conclusions:**

Memantine did not improve visuospatial working memory of the patients with PD-MCI. Treatment for PD should be planned carefully considering the impact on cognitive function. Further study is needed to establish new therapeutic strategy.

**Trial registration:**

UMIN000046104. Retrospectively registered.

First registration date: 28 Sept 2017.

## Introduction

Parkinson’s disease (PD) is a neurodegenerative disease characterized not only by motor symptoms. A cohort study reported that patients with PD had a six-fold greater risk of developing dementia compared with that in normal individuals [1]. A meta-analysis reported that the prevalence of mild cognitive impairment in PD (PD-MCI) was 26% [2]. In further, Janvin et al. reported that the prevalence was 55% in a patient group with mean disease duration of over 10 years [3]. Another study reported that 59% of patients with persistent PD-MCI for 1 year had converted to dementia during the follow-up period [4]. These reports indicate that persistent cognitive impairment of the patients with PD represents increased risk of dementia.

With regards to cognitive impairment in PD, various studies have reported impairments in frontal executive function including working memory [5]. Working memory is responsible for the short-term storage and online manipulation of information necessary for higher cognitive function; impaired working memory can disrupt activities of daily living. One major test to assess working memory is the n-back test, which was developed in the 1950s by Kirchner. Briefly, subjects are presented a sequence of stimuli one-by-one. They must decide and react immediately if the currently presented stimulus is identical to that presented N trials ago. Many neuroimaging studies have used the verbal n-back task during functional MRI (fMRI) to explore brain activation associated with working memory processing, and n-back task performance is associated with the activations of bilateral frontal and cortical regions [6]. In further, task performance of working memory is associated with dopaminergic neurotransmission in the striatum [7, 8]. Later, a visuospatial n-back test was developed in fMRI studies to assess visuospatial working memory in normal subjects [9, 10]. This test has a merit that the impact of verbal ability is smaller than another cognitive test, and it can assess both visuospatial recognition and working memory in a single test. Recently, we have reported that combinations of functional neuroimaging and the visuospatial n-back test are beneficial to evaluate the impaired visuospatial working memory of the patients with PD [11]. Furthermore, fMRI has an advantage to explore medication-related changes of blood-oxygen-level-dependent (BOLD) signal in enhancement or reduction of cortical activations during performing cognitive task. Using fMRI, the pharmacological effect of cholinesterase inhibitors for Alzheimer’s disease (AD) [12], or that of a peripheral inhibitor of the enzyme catechol-*O*-methyltransferase for PD [13], have been studied and detected as significant changes of BOLD signal in comparison with placebo.

Focusing on the pathological features of dementia in PD (PDD) and dementia with Lewy bodies, both are related to the accumulation of diffuse alfa-synuclein aggregates and Alzheimer-type changes [14]. In addition, striatal glutamatergic hyperactivity in an animal model of parkinsonism was reported [15]. Several studies have reported efficacy of cholinesterase inhibitors for PDD [16, 17]. However, cholinesterase inhibitors have an adverse effect of increasing risk of agitation, and these were not more effective than placebo in treating agitation in the patients with AD [18, 19]. Another anti-dementia drug for Alzheimer’s disease (AD) is memantine (N-methyl d-aspartate receptor antagonist) which targets glutamatergic neurotransmission. For the treatment of AD, meta-analyses have proven that memantine had a better outcome than those receiving placebo, and that memantine was not associated with a significant frequency of adverse events [20, 21]. There has been also positive effect of memantine for PDD, showing that memantine improved global clinical status or behavioral symptoms [22]. However, therapeutic efficacy of memantine for the patients with PD-MCI has not been established.

Therefore, we aimed to investigate whether memantine can alter brain function of the patients with PD-MCI, using fMRI. We explored the brain regions associated with visuospatial working memory, and searched the differences in behavioral performance and neuroimaging findings between memantine intervention and placebo. We hypothesized that specific changes of BOLD signals were detected in association with enhancement or reduction of brain activations as a pharmacological influence of memantine.

## Materials and methods

### Participants

We enrolled 12 right-handed patients with PD-MCI from the Department of Neurology at Nagoya City University Hospital. The study was approved by the local Ethical Committee and complied with national legislation and the Declaration of Helsinki guidelines. All patients provided written informed consent prior to data acquisition. None had any disease affecting motor and cognitive functions except for PD. All patients fulfilled the clinical diagnostic criteria according to United Kingdom Parkinson’s Disease Brain Bank Criteria for clinical diagnosis [23]. PD-MCI was diagnosed according to the Level II criteria of the Movement Disorder Society Task Force which advocated the detection thresholds of − 2 SD had significant impact on the discriminative validity of all measures [24, 25]. We excluded patients if they had depression, severe hearing loss, or any other disease that might severely influence data collection. We also excluded patients if they had dementia according to the criteria for PD dementia provided by the Movement Disorder Society Task Force [26]. Two patients withdrew from study. Hence, the population in this study constitutes the 10 patients (eight males and two females) who completed follow-up.

### Neuropsychological tests and clinical assessments

Movement disorder specialists did the complete clinical assessments for all participants. The motor section of Unified Parkinson’s Disease Rating Scale (UPDRS part3) was used to assess severity of motor symptoms [27]. Epworth Sleepiness Scale (ESS) was used to assess daytime sleepiness [28]. The dose of dopamine agonists (DA) was normalized using an L-DOPA equivalent daily dose of dopamine agonists (LEDD) [29].

Global cognitive function was assessed with MMSE and Montreal Cognitive Assessment (MoCA). Psychomotor speed and attention were assessed with Trail-Making Test Part A (TMT-A) and Paced Auditory Serial Addition Test (PASAT). Executive function and rapid set shifting were assessed with Trail-Making Test Part B (TMT-B). Visuospatial function was assessed using visuospatial version of the 0-back test, while visuo-spatial working memory was accessed using visuospatial version of the 1-back and 2-back test. PD-MCI was diagnosed according to the Level II criteria of the Movement Disorder Society Task Force [24]. In accordance with this criteria, PD-MCI was defined when patients’ scores were 2 SD below the normative mean score of the neuropsychological assessments, and it was defined when their impairment on at least two tests represented by either two impaired tests in one cognitive domain (single domain impairment) or one impaired test in two different cognitive domains (multi domain impairment) [25]. Of the 10 patients with PD-MCI, 8 patients had impairments in multiple cognitive domains.

### Visuospatial n-back test

The n-back test used in this study is a modified version of visuospatial n-back test which was reported in fMRI studies for normal subjects [10]. The details of this test have been reported in our previous paper [11]. In summary, the patients were asked to perform the tests with 3 load levels during the fMRI. The stimuli were white squares randomly presented in 1 of 8 spatial locations on a screen, through a mirror positioned on a head-coil. The presentation of the stimuli was controlled by a program (Presentation software) that initiated the acquisition of the MRI and the behavioural data. For the 0-back test, the subjects were instructed to press the left button with their index finger when a white square was presented. For the 1-back test, they were instructed to press the left button whenever a stimulus was presented in the same location as the previous stimulus. For the 2-back test, they had to press the left button whenever a stimulus was presented in the same location as the two trials previous. When the stimulus was presented in any other location during the 1-back and 2-back test, the patients were instructed to press the right button with the middle finger. The higher the number n requires the higher level of attention and visuospatial working-memory.

### Imaging protocol

**Fig. 1:**
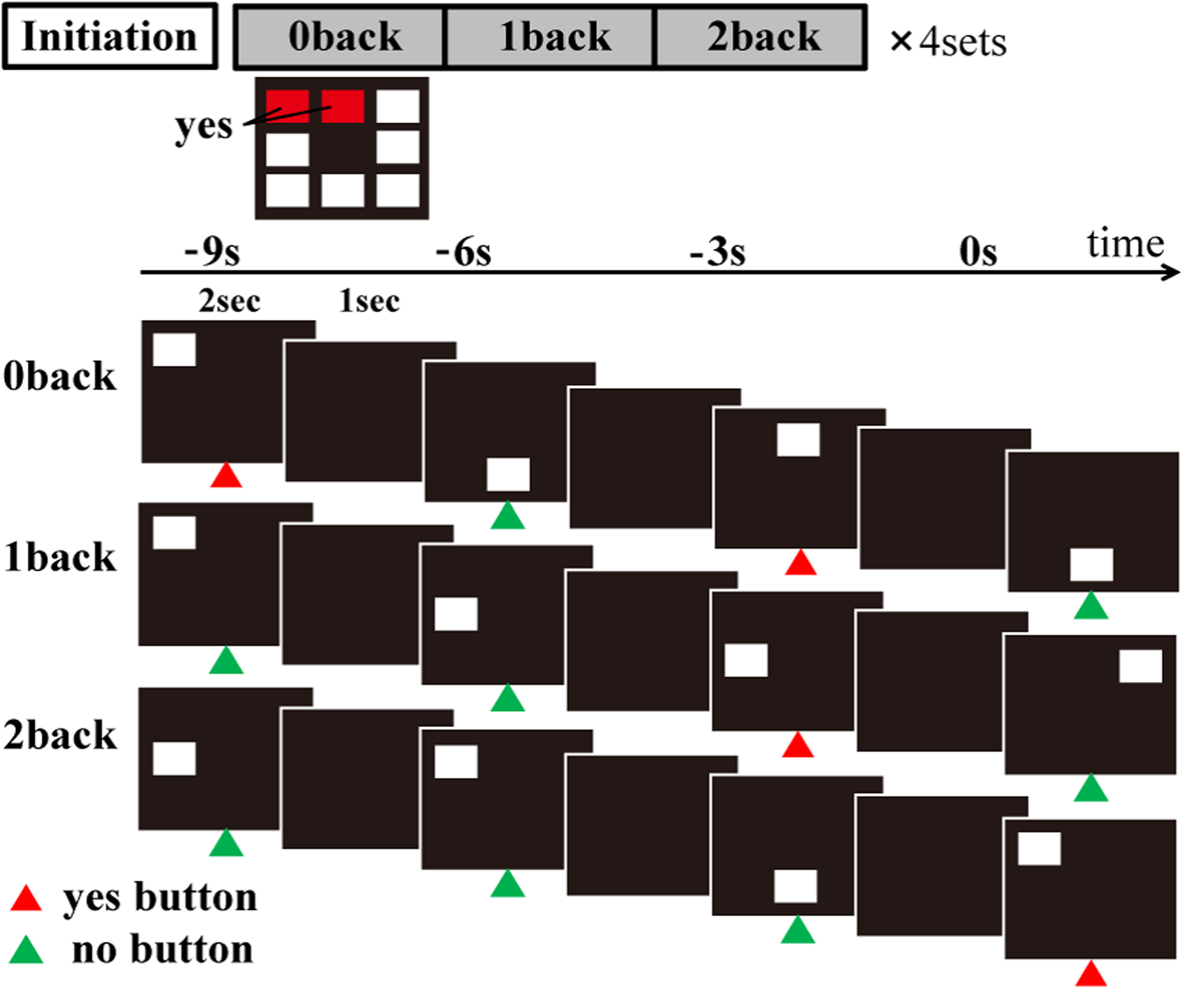
Visuospatial n-back test

To detect brain regions activated by n-back test, we used a block-design protocol, which alternated between test and rest conditions. The details of imaging protocol have been reported in our previous paper [11]. For test conditions, the white square was randomly presented for 2 second in 1 of 8 possible locations on screen; a black screen was presented for 1 second after stimulus presentation. Each test condition consisted of 15 trials over the course of 45 second; each rest condition lasted for 15 second. During a single scan, each test condition was repeated 4 times, in numerical order (0–1-2 back). Thus, each condition included 60 trials (Fig. 1). The test was performed during the ‘On’ state to avoid cognitive change or anxiety arising from being in the ‘Off’ state during testing.

The figure shows the protocol of visuospatial n-back test. In task conditions, the white square was presented for 2 s at random in 1 of 8 possible locations on screen, and black screen was presented for 1 s after the presentation of stimuli. In the 0-back test, the subjects were instructed to press the left button with their index finger when the white square was presented in predetermined locations. They were instructed to press the right button when the stimulus appeared in any other location. In the 1-back test, the subjects were instructed to press the left button when the stimulus presented in the same location as the previous one. In the 2-back test, they were instructed to press the left button whenever a stimulus was presented in the same location as the two trials previous. When the stimulus was presented in any other location during the 1-back and 2-back test, the patients were instructed to press the right button with the middle finger. The details of visuospatial n-back test have been reported in our previous paper [11].

### Study design

**Fig. 2:**
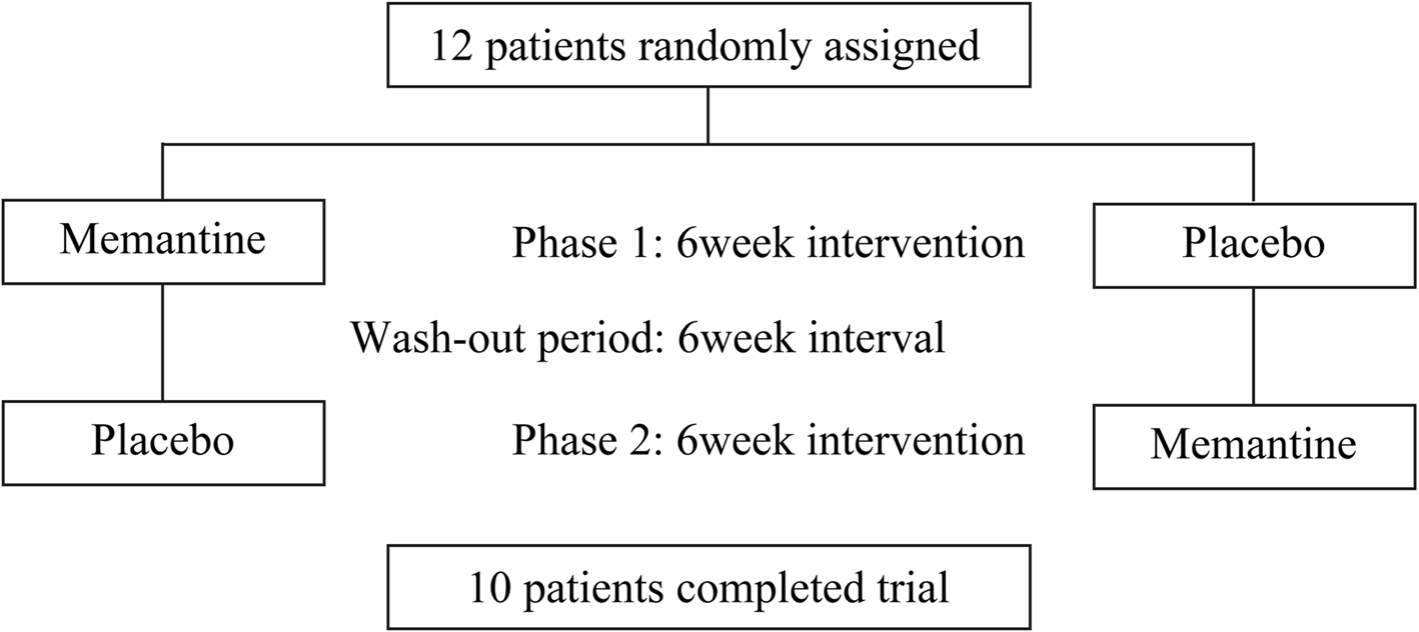
Study design

The study followed a randomized double-blinded crossover protocol. Patients were randomly assigned to either the memantine or placebo groups using a computer-based random number table. During the first study period, the patients in the memantine group were given memantine at 5 mg/day in the first week, and the dose was increased by 5 mg/day per week, with the final dose of 20 mg/day from the fourth week to the sixth week. The maximum dose duration was 3 weeks in the intervention period. The patients in the placebo group were given a placebo following the same regimen as memantine. During the maximum dose period, fMRI scanning and neuropsychological tests were performed. After the first study period, patients underwent 4 weeks of washout period. The washout duration was based on the previous studies using the prospective crossover protocol for memantine [30, 31]. Following the crossover protocol, patients who received memantine in the first intervention were administered a placebo in the second intervention, and vice versa. fMRI and neuropsychological tests were repeated during the 3 weeks of maximum dose administration in the second study period. Patients were requested to retain the same medication regime, regardless of memantine or placebo, until the study ended. They were unaware of the protocol assignment until the end of study (Fig. 2).

The figure shows study design. After inclusion evaluation, patients were randomly assigned to either memantine or placebo during the first study period. After the first study period, they underwent 4 weeks of washout period. Following the crossover protocol, patients who received memantine in the first intervention were administered a placebo in the second intervention, and vice versa.

### Image data acquisition and analysis

The methods of image data acquisition and analysis have also been described in our previous report [11]. All MRI were acquired with a Siemens Skyra syngo MR E11 3.0 T scanner (Siemens, Germany). High-resolution T1-weighted images were acquired via volumetric 3D spoiled gradient recall sequence. Acquisition parameters were as follows: repetition time (TR) = 1900 ms, echo time (TE) = 2.43 ms, flip angle (FA) = 9, field of view (FOV) = 256 × 256 mm, slice thickness = 1 mm, slice gap = 0, voxel size = 1 × 1 × 1 mm, number of slices = 176. The fMRI measurements were performed using a gradient echo EPI sequence: repetition time (TR) = 2500 ms, echo time (TE) = 30 ms, slice thickness = 3 mm, total 149 volumes, with matrix size of 64 × 64 and field of view of 192 × 192 mm, resulting in voxel size of 3 × 3 × 3 mm. All the images were pre-processed and analysed with Matlab (version 8.1, The Mathworks Inc., Natick, MA) and SPM8 software (Department of Cognitive Neurology, London). Images were realigned to correct for movement and normalised to Montreal Neurologic Institute (MNI) space. The transformed image data were smoothed with a Gaussian philtre (full width at half-maximum = 10 mm).

The image data were analysed with a random effect procedure and a parametric model to identify the brain areas where the activation correlated with the task. We specified the first level analysis model, estimated and defined the parameters and t-contrasts for n-back test conditions vs. the rest condition. The resulting contrast maps from each contrast and for each subject were then used in a second level random effects analysis for between groups effect (memantine vs. placebo). Group comparison was performed for three loads of the n-back test condition. The statistical significance was determined at *P* < 0.01 (uncorrected) with cluster size > 50 voxels in analysis.

### Statistical analysis

To investigate statistical differences of the clinical and neuropsychological data between memantine intervention and placebo, each data was compared using the independent t-tests as appropriate. The scores and reaction times of n-back test were analysed using one-way analysis of variance, because each load level required several neuropsychological cognitive domains. SPSS version 15.0 was used for statistical analyzes. *P* < 0.05 was considered significant.

## Results

### Clinical and neuropsychological data of the patients with PD-MCI who completed follow-up

The clinical profiles and demographic data at the time of inclusion were summarised in Table 1. The table shows baseline data of each patient who completed the study. Mean age was 69.0 ± 2.7 years, duration from disease onset was 5.1 ± 2.9 years, UPDRS part 3 score was 17.0 ± 6.6, and MMSE was 26.5 ± 1.6. Nine patients received L-DOPA therapy; Four patients were treated with only L-DOPA; Six patients were treated with both DA and L-DOPA. We did not find any correlation between the neuropsychological test scores (MMSE, MoCA, and PASAT) and the dosage of L-DOPA or LEDD.

**Table 1:** Clinical profiles of the patients who completed follow-up

	Age (years)	Duration (years)	UPDRS part3	L-DOPA (mg)	LEDD (mg)
Patient 1	68	9	31	350	0
Patient 2	72	3	20	250	0
Patient 3	69	2	8	100	100
Patient 4	66	2	19	200	0
Patient 5	72	2	18	200	0
Patient 6	66	4	19	300	75
Patient 7	69	7	17	400	75
Patient 8	74	8	8	150	50
Patient 9	68	9	17	200	150
Patient 10	67	5	13	0	135
Mean ± SD	69.0 ± 2.7	5.1 ± 2.9	17.0 ± 6.6	215.0 ± 118.0	58.5 ± 58.0
	MMSE	MoCA	PASAT	TMT-A (sec)	TMT-B (sec)
Patient 1	25	20	21	79	420
Patient 2	29	24	28	69.7	238
Patient 3	27	25	28	39.5	159
Patient 4	28	21	19	48.4	151.4
Patient 5	29	23	32	77.4	194.4
Patient 6	26	17	18	100.6	379.8
Patient 7	25	18	18	65.4	420
Patient 8	26	19	16	53.6	107
Patient 9	25	18	8	56.8	167.3
Patient 10	25	16	26	104.7	357
Mean ± SD	26.5 ± 1.6	20.1 ± 3.1	21.4 ± 7.1	69.5 ± 21.4	259.4 ± 121.9

*Duration* Disease duration, *UPDRS part3* motor sections of United Parkinson’s Disease Rating Scale, *L-DOPA* dosage of L-DOPA, *LEDD* L-dopa equivalent daily dose of dopamine agonist, *MoCA* Montreal Cognitive Assessment, *PASAT* Paced Auditory Serial Addition Test, *TMT-A* time to complete part A of the Trail Making Test, *TMT-B* time to complete part B of the Trail Making Test, *sec* seconds

### Clinical and neuropsychological data in memantine intervention and placebo

The clinical and neuropsychological findings resulting from memantine intervention and placebo were summarized in Table 2. The dose of memantine was finally increased up to 20 mg per day for 10 patients who completed follow-up. There was no significant difference in the scores of UPDRS part3 between memantine and placebo. There were no significant differences in the total scores of MMSE, MoCA, PASAT, and the time to complete TMT-B. However, the time to complete TMT-A was significantly longer in the memantine comparing with placebo (*P* < 0.05). In addition, we found a trend that the score of ESS during memantine intervention was higher than placebo (*P* = 0.07).

**Table 2:** Clinical and neuropsychological data in memantine intervention and placebo

	Placebo	Memantine	***P*** value
UPDRS part3	17.0 ± 6.6	18.0 ± 5.2	N.S.
ESS	7.1 ± 3.8	8.9 ± 5.0	*P* = 0.07
MMSE	26.1 ± 2.2	25.4 ± 2.5	N.S.
MoCA	20.1 ± 3.1	18.7 ± 4.1	N.S.
PASAT	21.4 ± 7.1	19.8 ± 6.7	N.S.
TMT-A	69.5 ± 21.4	86.6 ± 38.5	*P* < 0.05
TMT-B	259.4 ± 121.9	312.9 ± 132.6	N.S.
0-back test (number)	17.1 ± 2.3	16.3 ± 4.1	N.S.
1-back test (number)	14.9 ± 3.4	13.9 ± 2.9	N.S.
2-back test (number)	12.4 ± 4.6	10.2 ± 3.9	*P* < 0.05
0-back test (seconds)	790.3 ± 155.4	800.6 ± 179.1	N.S.
1-back test (seconds)	991.7 ± 171.5	1015.4 ± 208.7	N.S.
2-back test (seconds)	1087.4 ± 208.7	1167.9 ± 274.0	N.S.

This table shows the comparison of neuropsychological findings between memantine intervention and placebo. ESS: Epworth Sleepiness Score; 0-back test (number): number of correct answers in 0-back test (minimum 0, maximum 20); 1-back test (number): number of correct answers in 1-back test (minimum 0, maximum 20); 2-back test (number): number of correct answers in 2-back test (minimum 0, maximum 20); 0-back test (seconds): reaction time of 0-back test; 1-back test (seconds): reaction time of 1-back test; 2-back test (seconds): reaction time of 2-back test; *N.S.* not significant

In comparisons for behavioral performance between memantine and placebo, there were no significant differences in the reaction time and the number of correct answers of the 0-back and 1-back tests. However, number of correct answers of 2-back test during memantine intervention was significantly worse than placebo (memantine, 10.2 ± 3.9; placebo, 12.4 ± 4.6; *P* < 0.05).

### fMRI data

**Fig. 3:**
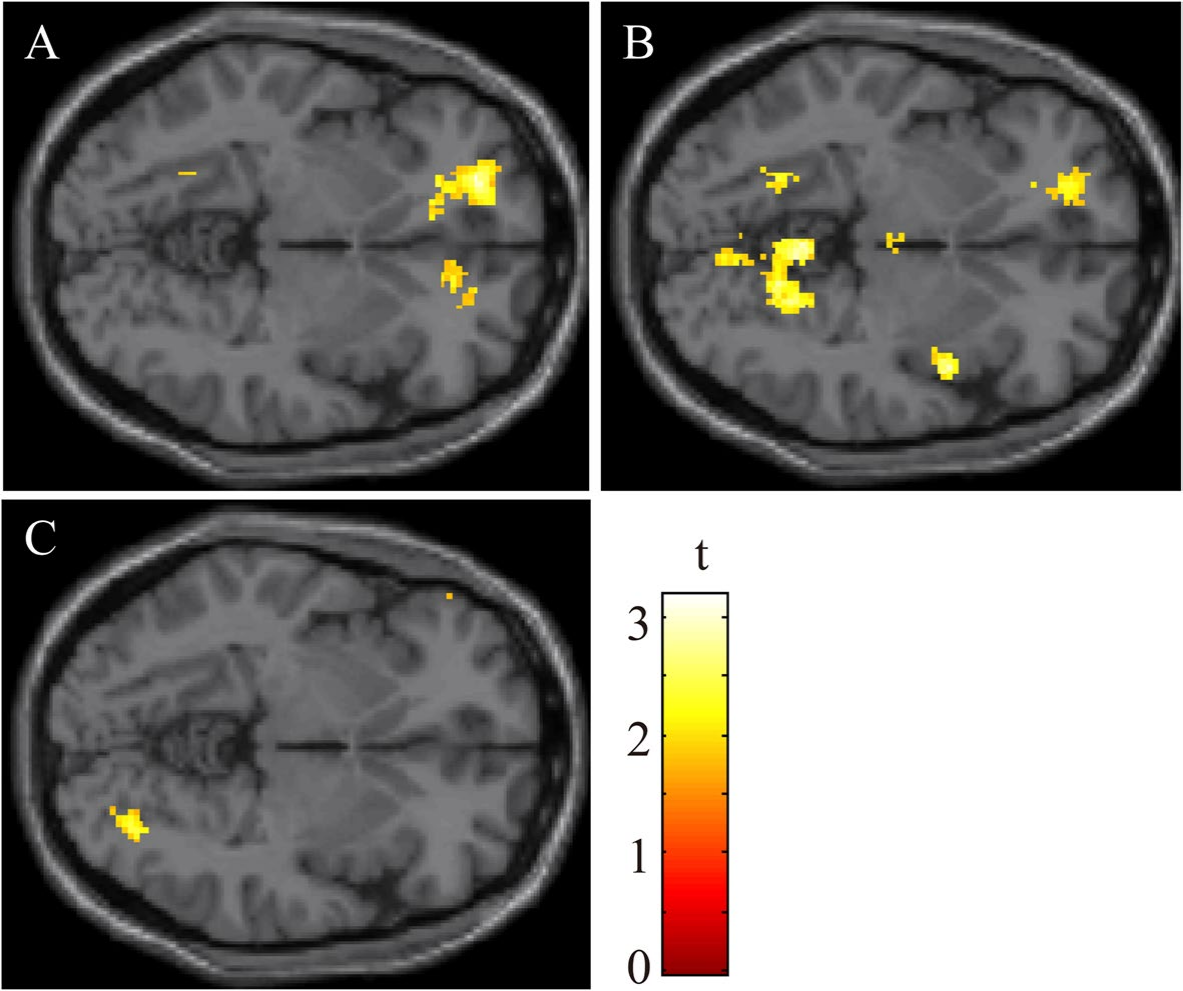
fMRI analyses comparing memantine and placebo

To investigate whether memantine can alter brain function of the patients with PD-MCI, we searched the significant differences in fMRI findings between memantine intervention and placebo. The brain regions shown in Fig. 3 were volumes where the t-contrast for decrease and increase. There were no significant regions enhanced by memantine compared with placebo at any load of the n-back tests. In contrast, when exploring the regions reduced by memantine intervention comparing with placebo, we found significant deactivations within the right lingual gyrus (LG) and left superior frontal gyrus (SFG) in comparison between the 2-back versus 0-back test. The result of 2-back test showed significantly reduced activations within the right superior temporal gyrus and left SFG during memantine intervention, while we did not find any specific findings within frontotemporal or parietal lobes in the results of 0-back, 1-back, and the 1-back versus 0-back test.

The figure shows the results of fMRI analyses comparing memantine and placebo. The colour-bar represents t values as reference. All the images presented at *P* < 0.01 (uncorrected) with cluster size > 50 voxels in analysis. (A) The coloured regions show the change of brain activation during memantine intervention compared with placebo in the 2-back test. We found significantly reduced activation within the right superior temporal gyrus and left superior frontal gyrus (SFG). (B) The image shows significantly reduced activation within the right lingual gyrus and left SFG during memantine intervention in comparison between the 2-back versus 0-back test. (C) The image shows the enhanced regions during memantine intervention compared with placebo in the 2-back test. We did not find any specific findings in frontotemporal or parietal lobes.

## Discussion

To our knowledge, neuroimaging studies to explore the changes in brain activation during memantine anti-dementia therapy in patients with PD-MCI have never been conducted. Therefore, the present study is the first pilot study that aimed to investigate whether memantine can alter brain function associated with the visuospatial working memory of patients with PD-MCI. Using a combination of visuospatial n-back test and fMRI, we revealed that memantine made worse local brain function within the right LG and left SFG, comparing with placebo. In contrast, we did not find any specific findings enhanced by memantine.

### Memantine reduce brain activations associated with working memory in the superior frontal gyrus and lingual gyrus

There were no significant regions enhanced by memantine compared with placebo at any load of the n-back tests. In contrast, fMRI findings of the 2-back versus 0-back test during memantine intervention showed a reduction of brain activations in the right LG and left SFG. We did not find any specific regions in the results of 0-back, 1-back, and 1-back versus 0-back test. In behavioral analysis, the number of correct answers of 2-back test during memantine intervention was worse than placebo.

Generally, the changes of task-related brain activation were most prominent for the 2-back condition; subtracting the activated regions in the 0-back test (the easiest condition) from those in the 2-back test is adequate for the purpose of searching brain regions associated with working memory. The subtracting condition of the 0-back test from those of the 1-back test had limited statistical power to detect brain areas associated with working memory because the 1-back test requires low-grade visuospatial working memory. Considering these, the deactivations within the right LG and left SFG may associate with an impaired visuospatial working memory resulting from memantine intervention.

Concerning the observed regions, it was reported that impaired visual memory was related to either damage to the region or disconnections between the LG and other brain structures [32]. Mangun et al. reported that activation of the LG has been shown in association with selective visual spatial attention in their fMRI study [33], and Machielsen et al. reported that LG has been linked to encoding of complex images [34]. Therefore, the deactivation of the LG in the present study may be related to worse visuospatial working memory in patients with PD-MCI during memantine intervention compared with the placebo.

Second, concerning the role of SFG, Carlson et al. reported that the LG is dependent on the memory load of the visuospatial n-back task in fMRI [10]. In their study, which targeted normal volunteers, a comparison of the 2-back and 0-back tasks revealed bilateral activation in the medial frontal gyrus, superior frontal sulcus, and SFG. They also reported that a comparison between the 1-back and 0-back tasks showed activation in only a few brain areas. In our study, we did not detect any significant difference in the activation of the medial frontal gyrus, between the 2-back versus 0-back test. One explanation may be that fMRI findings which associated with working memory are likely to be influenced by daytime sleepiness in patients with PD-MCI.

In neuropsychologically, the correct answer rate of the 2-back test during memantine intervention was worse than that of the placebo. Although not statistically significant, we found a trend that a reaction time of the 2-back test and the score of ESS were worse during the memantine intervention than that of the placebo. These consistent trends suggest the possibility that memantine deteriorates both working memory and daytime sleepiness in patients with PD-MCI. Several fMRI studies have reported that sleepiness is related to degrades of working memory performance [35, 36]. Using resting state fMRI, Li et al. have reported that functional connectivity between the superior parietal lobule and right superior frontal gyrus (dorsolateral prefrontal cortex) of patients with primary insomnia was significantly poorer than that of the control group [37]. Considering these, The deactivation within the SFG during the memantine intervention in our study supports the conclusion that memantine enhances daytime sleepiness but reduces visuo-spatial working memory performance in patients with PD-MCI. .

### Clinical implications for the treatment of PD-MCI

The development of dementia in the patients with PD is caused by dual pathology, that is, Diffuse Lewy body and AD pathology [14]. First, considering the Diffuse Lewy body pathology, therapeutic choice for cognitive impairment in PD is L-DOPA and DA, because dopamine may improve the neuro-transmission of cortico-striatal circuit. Some studies have reported the efficacy of L-DOPA or DA for cognitive impairments in patients with PD [38, 39]. Concerning the AD pathology, several studies have reported the efficacy of cholinesterase inhibitors for PDD [16, 17], and the efficacy of memantine for PDD [22]. However, neuropathological research on PD-MCI is limited, and therapeutic evidence of PD-MCI has not been established other than for cholinesterase inhibitors.

Focusing on the cognitive influence of memantine for the normal elderly, a double-blind pilot study which targeted for the participants of age-associated memory impairment (no dementia or MCI), reported positive effect, suggesting that memantine has a possibility to improve attentional processes if the memory impairment is in subtle stage [40]. Whereas, in the present study which targeted for PD-MCI, we found a negative influence: A time to complete TMT-A and number of correct answers of the 2-back test were worse during the memantine intervention. Generally, the variability of PD-MCI is associated with neurotransmitter abnormalities and genetic characteristics [41]. The dual syndrome hypothesis has been proposed two distinct genetic syndromes that influence executive function and memory/visuospatial abilities in patients with PD [42]. Furthermore, Lawrence et al. had reported that multiple domain cognitive impairment was more frequent than single domain impairment of the patients with PD (93% of the patients were identified as having multiple domain cognitive impairment) [43]. In our study, of the 10 patients with PD-MCI, 8 patients had impairments in multiple cognitive domains. Therefore, multiple cognitive impairments representing the heterogenous pathology of PD-MCI may influence neuroimaging results associated with the visuospatial working memory. Furthermore, the increased daytime sleepiness caused by memantine may have worsened the visuospatial working memory performance in patients with PD-MCI.

It is well known that dopamine agonists tend to increase daytime sleepiness and the risk of sleep attacks in patients with PD [44]. In the present study, all patients received the maximum dose of memantine during the study period, and six of the patients were treated with both dopamine agonists and L-DOPA. Therefore, it is plausible that the combination of dopamine agonists and a maximum dose of memantine may have enhanced daytime sleepiness resulting in a reduction in psychomotor speed and attention. A recent review which investigated the efficacy of anti-dementia drugs for PDD, reported that cholinesterase inhibitors, but not memantine, improved cognitive function [45]. Considering these findings, the maximum dose of memantine is not beneficial for the patients with PD-MCI. In addition, the results of present study indicate that treatment for PD should be planned carefully considering the impact on cognitive function.

### Limitations and strengths

This fMRI study has some limitations. First, the main limitation was the small sample size of this study. The statistical power to search differences in behavioral performance and neuroimaging findings was restricted. However, despite small sample size, we found the number of correct answers of 2-back test during memantine intervention was worse than that of placebo. In further, we found a tendency that the reaction time of 2-back test during memantine intervention was worse than that of placebo. Considering these behavioral differences, we concluded that memantine did not improve working memory in patients with PD-MCI. To clarify how memantine influences cognitive function from a long-term perspective, further study with larger populations and a longer follow-up is needed. As for the smoothing kernel in fMRI analysis, although there is no easy answer regarding how much we should smooth imaging data, the main downside to higher smoothing is the loss of spatial specificity. Because of the small sample size, we had to reduce noise as much as we can using relatively high smoothing kernel compared with voxel dimension. Therefore, we used the 10 mm smoothing kernel instead of the reduction of spatial resolution. Because of these reasons, we could not find significant voxels in imaging analysis with the family-wise error correction. Second, the results of n-back test were likely to be influenced by variety of patients’ profile such as the doses of L-DOPA and LEDD of DA. Simioni et al. reported that administration of L-DOPA remediated the working memory deficit in the patients with PD and resulted in a different pattern of performance-correlated activity of the fMRI findings compared to off dopamine replacement therapy [46]. Thus, it was possible that L-DOPA may influence neuroimaging findings. In our study, we assumed that the fMRI findings were not significantly influenced by L-DOPA because the dosage of L-DOPA was the same during the memantine intervention and the placebo period, due to our study design. In further, L-DOPA dose did not affect behavioural performance directly, because there were no correlations between the scores of neuropsychological tests and the dosage of L-DOPA. Third, there is also another possibility that neuroimaging results of n-back tests were affected by negative emotion including anxiety and depression, because negative emotion has a particularly worsen selectivity of attention as well as motivating action and behavior. To reduce the difference of negative emotion in each patient, we performed fMRI scanning during the ‘On’ state, because it was predicted that anxiety arising from being in the ‘Off’ state might influence more compared with ‘On’ state. However, there is no specific way to quantify real influence of emotions during cognitive task, and it may be a fundamental technical limitation of the activation study using fMRI.

However, the present study has some strengths and novel aspects. First, this is the first neuroimaging study which identified the changes of brain activation associated with memantine in patients with PD-MCI. Second, we stress that the pharmacological fMRI protocol has a potential to explore the influence of drugs for central nervous system noninvasively. To clarify the efficacy and pharmacological influence for central nervous system of another anti-Parkinson or anti-dementia therapy, the accumulation of neuroimaging findings is necessary.

## Conclusions

We revealed that memantine reduced functional brain activation in the right LG and left SFG in patients with PD-MCI. Memantine did not improve working memory of the patients with PD-MCI. Treatment for PD should be planned carefully considering the impact on cognitive function. Further study is needed to establish new therapeutic strategy for the cognitive impairment in patients with PD.

## Data Availability

The datasets used in this study are available from the corresponding author on reasonable request.

## Abbreviations

fMRI: Functional magnetic resonance imaging
PD: Parkinson disease
PDD: PD with dementia
PD-MCI: PD with mild cognitive impairment
AD: Alzheimer’s disease
BOLD: Blood-oxygen-level-dependent
UPDRS: Unified Parkinson’s Disease Rating Scale
DA: Dopamine agonists
LEDD: L-DOPA equivalent daily dose of dopamine agonists
MoCA: Montreal Cognitive Assessment
TMT-A: Trail-Making Test Part A
TMT-B: Trail-Making Test Part B
PASAT: Paced Auditory Serial Addition Test
AVLT: Auditory Verbal Learning Test
SFG: Superior frontal gyrus
LG: Lingual gyrus

## References

[1] Aarsland D, Andersen K, Larsen JP, Lolk A, Nielsen H, Kragh-Sørensen P (2001). Risk of dementia in Parkinson’s disease: a community-based, prospective study. Neurology.

[2] Aarsland D, Bronnick K, Williams-Gray C, Weintraub D, Marder K, Kulisevsky J (2010). Mild cognitive impairment in Parkinson disease: a multicenter pooled analysis. Neurology.

[3] Janvin C, Aarsland D, Larsen JP, Hugdahl K (2003). Neuropsychological profile of patients with Parkinson’s disease without dementia. Dement Geriatr Cogn Disord.

[4] Pedersen KF, Larsen JP, Tysnes O-B, Alves G (2017). Natural course of mild cognitive impairment in Parkinson disease: a 5-year population-based study. Neurology.

[5] Lee JE, Cho KH, Song SK, Kim HJ, Lee HS, Sohn YH (2013). Exploratory analysis of neuropsychological and neuroanatomical correlates of progressive mild cognitive impairment in Parkinson's disease. J Neurol Neurosurg Psychiatry.

[6] Hui A, He W, Wu J, Junjun Z, Zhenlan J, Li L (2019). A coordinate-based meta-analysis of the n-back working memory paradigm using activation likelihood estimation. Brain Cogn.

[7] Landau SM, Lal R, O’Neil JP, Baker S, Jagust WJ (2009). Striatal dopamine and working memory. Cereb Cortex.

[8] Backman L, Nyberg L, Soveri A, Johansson J, Andersson M, Dahlin E (2011). Effects of working-memory training on striatal dopamine release. Science.

[9] Courtney SM, Petit L, Maisog JM, Ungerleider LG, Haxby JV (1998). An area specialized for spatial working memory in human frontal cortex. Science.

[10] Carlson S, Martinkauppi S, Rama P, Salli E, Korvenoja A, Aronen HJ (1998). Distribution of cortical activation during visuospatial n-back tasks as revealed by functional magnetic resonance imaging. Cereb Cortex.

[11] Shoji K, Yoko S, Yoshino U, Noriyuki M (2021). Impairment of the visuospatial working memory of the patients with Parkinson’s disease. BMC Neurol.

[12] McGeown WJ, Shanks MF, Forbes-McKay KE, Waiter GD, Elrick I, Venneri MG (2010). Established donepezil treatment modulates regional brain activation in early Alzheimer’s disease. Curr Alzheimer Res.

[13] Tambasco N, Muti M, Chiarini P, Tarducci R, Caproni S, Castorioto A (2014). Entacapone reduces cortical activation in Parkinson’s disease with wearing-off: a f-MRI study. PLoS One.

[14] Irwin DJ, White MT, Toledo JB, Xie SX, Robinson JL, Van Deerlin V (2012). Neuropathologic substrates of Parkinson disease dementia. Ann Neurol.

[15] Starr MS (1995). Glutamate/dopamine D1/D2 balance in the basal ganglia and its relevance to Parkinson’ disease. Synapse.

[16] Rolinski M, Fox C, Maidment I, Mcshane R (2012). Cholinesterase inhibitors for dementia with Lewy bodies, Parkinson’s disease dementia and cognitive impairment in Parkinson's disease. Cochrane Database Syst Rev.

[17] Ballard C, Kahn Z, Corbett A (2011). Treatment of dementia with Lewy bodies and Parkinson's disease dementia. Drugs Aging.

[18] Robert JH, Edmund J, Clive GB, Peter B, Richard GB, Roger B (2007). Donepezil for the treatment of agitation in Alzheimer's disease. N Engl J Med.

[19] Clive B, Marisa ML, Edmund J, Simon D, Alan S, Alan T (2005). Quetiapine and rivastigmine and cognitive decline in Alzheimer's disease: randomised double blind placebo controlled trial. BMJ.

[20] Reisberg B, Doody R, Stiffler A, Schmitt F, Ferris S, Jorg H (2003). Memantine in moderate-to-severe Alzheimer's disease. N Engl J Med.

[21] Shinji M, Taro K, Nakao I (2015). Memantine Monotherapy for Alzheimer’s disease: a systematic review and Meta-analysis. PLoS One.

[22] Emre M, Tsolaki M, Bonuccelli U, Destée A, Tolosa E, Kutzelnigg A (2010). Memantine for patients with Parkinson’s disease dementia or dementia with Lewy bodies: a randomised, double-blind, placebo-controlled trial. Lancet Neurol.

[23] Hughes AJ, Ben-Shlomo Y, Daniel SE, Lees AJ (1992). What features improve the accuracy of clinical diagnosis in Parkinson's disease: a clinicopathologic study. Neurology..

[24] Goldman JG, Holden S, Bernard B, Ouyang B, Goetz CG, Stebbins GT (2013). Defining optimal cutoff scores for cognitive impairment using Movement Disorder Society task force criteria for mild cognitive impairment in Parkinson's disease. Mov Disord.

[25] Litvan I, Goldman JG, Tröster AI, Schmand BA, Weintraub D, Petersen RC (2012). Diagnostic criteria for mild cognitive impairment in Parkinson’s disease: movement disorder society task force guidelines. Mov Disord.

[26] Emre M, Aarsland D, Brown R, Burn D, Duyckaerts C, Mizuno Y (2007). Clinical diagnostic criteria for dementia associated with Parkinson's disease. Mov Disord.

[27] Fahn S, Elton RI, Fahn S, Marsden CD, Calne DB, Goldstein M, Members of the UPDRS Development Committee (1987). Unified Parkinson’s disease rating scale. Recent Developments in Parkinsons Disease.

[28] Johns MW (1991). A new method for measuring daytime sleepiness: the Epworth sleepiness scale. Sleep.

[29] Tomlinson CL, Stowe R, Patel S, Rick C, Gray R, Clarke CE (2010). Systematic review of levodopa dose equivalency reporting in Parkinson's disease. Mov Disord.

[30] Matthew J, Thurtell M, Anand C, Joshi M, Alice C, Leone D (2010). Cross-over trial of gabapentin and Memantine as treatment for acquired Nystagmus. Ann Neurol.

[31] Nikolajsen L, Gottrup H, Kristensen AG, Jensen TS (2000). Memantine (a N-methyl-D-aspartate receptor antagonist) in the treatment of neuropathic pain after amputation or surgery: a randomized, double-blinded, cross-over study. Anesth Analg.

[32] Bogousslavsky J, Miklossy J, Deruaz JP, Assal G, Regli F (1987). Lingual and fusiform gyri in visual processing: a clinico-pathologic study of superior altitudinal hemianopia. J Neurol Neurosurg Psychiatry.

[33] Mangun GR, Buonocore MH, Girelli M, Jha AP (1998). ERP and fMRI measures of visual spatial selective attention. Hum Brain Mapp.

[34] Machielsen WC, Rombouts SA, Barkhof F, Scheltens P, Witter MP (2000). fMRI of visual encoding: reproducibility of activation. Hum Brain Mapp.

[35] Nissen C, Kloepfer C, Feige B, Piosczyk H, Spiegelhalder K, Voderholzer U (2011). Sleep-related memory consolidation in primary insomnia. J Sleep Res.

[36] Walker MP (2008). Cognitive consequences of sleep and sleep loss. Sleep Med.

[37] Li Y, Wang E, Zhang H, Dou S, Liu L, Tong L (2014). Functional connectivity changes between parietal and prefrontal cortices in primary insomnia patients: evidence from resting-state fMRI. Eur J Med Res.

[38] Masahiro I, Hiroshi K, Satoshi U (2017). Can levodopa prevent cognitive decline in patients with Parkinson's disease?. Am J Neurodegener Dis.

[39] Livia B, Valentina P, Maria CM, Raffaele B, Cesare I, Paolo S (2013). The effect of dopamine agonists on cognitive functions in non-demented early-mild Parkinson's disease patients. Funct Neurol.

[40] Ferris S, Schneider L, Farmer M, Kay G, Crook T (2007). A double-blind, placebo-controlled trial of memantine in age-associated memory impairment (memantine in AAMI). Int J Geriatr Psychiatry.

[41] Cosgrove J, Alty JE, Jamieson S (2015). Cognitive impairment in Parkinson’s disease. Postgrad Med J.

[42] Kehagia AA, Barker RA, Robbins TW (2010). Neuropsychological and clinical heterogeneity of cognitive impairment and dementia in patients with Parkinson’s disease. Lancet Neurol.

[43] Lawrence BJ, Gasson N, Loftus AM (2016). Prevalence and subtypes of mild cognitive impairment in Parkinson's disease. Sci Rep.

[44] Paus S, Brecht HM, Koster J (2003). Sleep attacks, daytime sleepiness, and dopamine agonists in Parkinson’s disease. Mov Disord.

[45] Wang H-F, Yu J-T, Tang S-W, Jiang T, Tan C-C, Meng X-F (2015). Efficacy and safety of cholinesterase inhibitors and memantine in cognitive impairment in Parkinson’s disease, Parkinson's disease dementia, and dementia with Lewy bodies: systematic review with meta-analysis and trial sequential analysis. J Neurol Neurosurg Psychiatry.

[46] Simioni AC, Dagher A, Fellows LK (2017). Effects of levodopa on corticostriatal circuits supporting working memory in Parkinson’s disease. Cortex..

